# [Retracted] lncRNA ST8SIA6‑AS1 facilitates proliferation and invasion in liver cancer by regulating miR‑142‑3p

**DOI:** 10.3892/etm.2024.12627

**Published:** 2024-06-27

**Authors:** Yang Zhang, Yan Yang, Yi Zhang, Zhisu Liu

Exp Ther Med 22:1348, 2021; DOI: 10.3892/etm.2021.10783

Following the publication of this paper, it was drawn to the Editors’ attention by a concerned reader that certain of the cell migration assay data shown in Fig. 2E on p. 5 and Fig. 4E on p. 7 were strikingly similar to data appearing in different form in other articles written by different authors at different research institutes that had already been accepted for publication elsewhere prior to the submission of this paper to *Experimental and Therapeutic Medicine*. In view of the fact that the abovementioned data had already been submitted and accepted for publication previously, the Editor of *Experimental and Therapeutic Medicine* has decided that this paper should be retracted from the Journal. The authors were asked for an explanation to account for these concerns, but the Editorial Office did not receive a reply. The Editor apologizes to the readership for any inconvenience caused.

